# Efficiency of Epidermal Grafts in Chronic Wounds: A Retrospective Observational Study

**DOI:** 10.1002/hsr2.71252

**Published:** 2025-09-18

**Authors:** S. Bruyeres, G. Dumas, N. Zenati, S. Blaise

**Affiliations:** ^1^ Service de Médecine Vasculaire Grenoble University Hospital BP 217 Grenoble France; ^2^ Univ. Grenoble Alpes, Inserm U1300, HP2 Grenoble France

**Keywords:** chronic wound, epidermal grafts, grafting technique, treatment

## Abstract

**Background and Aims:**

Chronic wounds represent a major challenge in medical management, requiring effective therapeutic approaches to promote healing. In recent years, epidermal grafts have emerged as a promising therapeutic option to improve the healing process of chronic wounds. This device only allows ultrasuperficial grafts to be performed using a suction/suction mechanism with a dermo‐epidermal cleavage zone, unlike other superficial grafting techniques such as pellet or mesh grafts. There is little data in the literature regarding clinical studies because it is difficult to conduct comparative studies to evaluate the efficacy of grafts. The indications can be broad and the etiologies of the wounds varied, as we would like to illustrate. This retrospective study aims to evaluate the benefit of epidermal grafts in the treatment of chronic wounds.

**Method:**

A retrospective study was carried out over a period of 4 years, from 2019 to 2023. We used a so‐called “superficial” grafting technique using an ultra superficial epidermal grafts CelluTome™ Epidermal Harvesting System (KCI, an Acelity company, San Antonio, USA). The objectives of these epidermal grafts could be twofold: either for analgesic purposes or to cover the wound to accelerate the healing process. Patient demographics, wound characteristics, treatment modalities, number of grafts and healing outcomes were collected and analyzed.

**Results:**

A total of 28 patients with chronic wounds were included in the study; 23 “vascular” wounds were treated (82.2%), including 11 of venous origin (39.3%), 5 of arterial origin (17.8%) and 7 mixed ulcers (25%). No side effects have been reported. Patient follow‐up averaged 22.70 (19.52) weeks. For the 15 patients whose objective was healing, 4 were healed after 1 month after the last Cellutome®.

**Discussion:**

This retrospective study demonstrates the potential value of epidermal grafting as a treatment for seams in hard‐to‐heal wounds. Epidermal grafts offer a number of advantages in the field of wound healing, particularly in difficult, recalcitrant and multifactorial wounds. The ease with which these grafts can be performed (broad aetiological indications, ambulatory nature, no need for a complex technical platform, no need for anesthesia) makes them accessible to a large number of patients. Despite these advantages, the results of epidermal grafts in terms of efficacy are difficult to assess. We did not find any comparative studies between the different superficial grafts.

**Conclusion:**

This procedure undoubtedly deserves to be better known and more widely used to optimize the experiments, particularly with regard to heating times.

## 1 Introduction

Chronic wounds represent a major challenge in medical management, requiring effective therapeutic approaches to promote healing. In recent years, epidermal grafts have emerged as a promising therapeutic option to improve the healing process of chronic wounds. Ultra superficial epidermal grafts have been performed for several years in the context of therapeutic impasses in chronic ulcerations [1]. There are still unresolved points of knowledge, such as the rate of graft application or very long‐term efficacy [2]. This device only allows ultrasuperficial grafts to be performed using a suction/suction mechanism with a dermo‐epidermal cleavage zone, unlike other superficial grafting techniques such as pellet or mesh grafts. A skin graft can be proposed for both skin covering and/or analgesic purposes. This retrospective study aims to evaluate the benefit of epidermal grafts in the treatment of chronic wounds over a period of 4 years, in the Vascular Medicine department of Grenoble University Hospital to assess efficacy in terms of analgesia and/or healing with longitudinal follow‐up.

## 2 Methods

This monocentric, retrospective, longitudinal and observational study without a control group was performed to evaluate epidermal grafts in chronic wounds. The main objective is to retrospectively describe the population of patients treated with ultra superficial epidermal grafts CelluTome™ Epidermal Harvesting System (KCI, an Acelity company, San Antonio, USA) and in terms of analgesia and/or recovery in patients treated in the Vascular Medicine department of Grenoble University Hospital. The population studied is all patients treated in the Vascular Medicine department since 2019, the date of acquisition of the Cellutome® device in the establishment, until may 2023.

The primary endpoint was to describe the number of patients and the number of ultra superficial grafts performed per patient whatever their etiology. The secondary objective was to evaluate the efficacy of the graft in terms of either pain reduction or ulcer surface reduction (combined criterion) with the number of ulcers with post‐graft pain reduction and the number of ulcers with post‐graft surface reduction. Patient demographics, wound characteristics, treatment modalities, number of grafts and healing outcomes were collected and analyzed. The definition of healing was a complete epidermization of the entire surface of the wound.

## 3 Population Patients

Patients were all recruited as outpatients or conventional inpatients at the Vascular Medicine Department of Grenoble University Hospital, Grenoble, France. The inclusion criteria were patients with wounds of any aetiology (venous or arterial ulcers, necrotic angiodermatitis or diabetic foot) of at least 6 weeks duration, patients who consulted the vascular medicine department from 2019 to 2023, who had ulcers for more than 6 weeks, with an age upper 18 years. Wound size should be between 1 cm^2^ and less than 25 cm^2^. the maximum authorized size was linked to the maximum surface area of the heating plates used in the devices: either 2.5 cm by 2.5 cm or 5 cm by 5 cm.

Patients will be required to understand and be willing to participate in the trial. All patients needed to have previously undergone specific debridement treatment for their wound with clean, healthy granulating bed. Patients could have had previous epidermal grafts.

The exclusion criteria were patients objecting to the use of their data for research purposes, clinicaly infected wound, unsuitable for split‐thickness skin grafting, necrotic or predominantly fibrinous ulcer, overly exudative ulcer, patients suffering from psychiatric pathologies with risk of agitation during the procedure, patients unable to remain motionless for at least 1 h.

## 4 Interventions and Wound Bed Preparation

**Figure 1:**
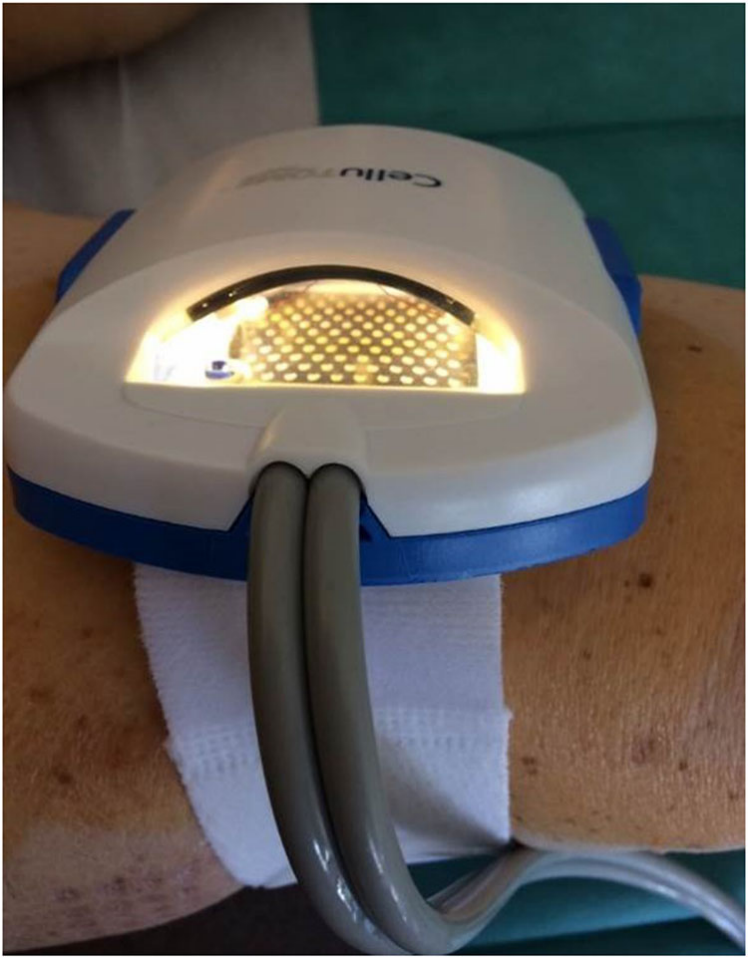
The suction head of the CelluTome Epidermal Harvesting System® device was applied to the thigh of the patient (donor site, generally the lateral or anterior surface of the thigh, homolateral to the grafted ulcer) for 40 min to harvest the epidermal graft.

**Figure 2:**
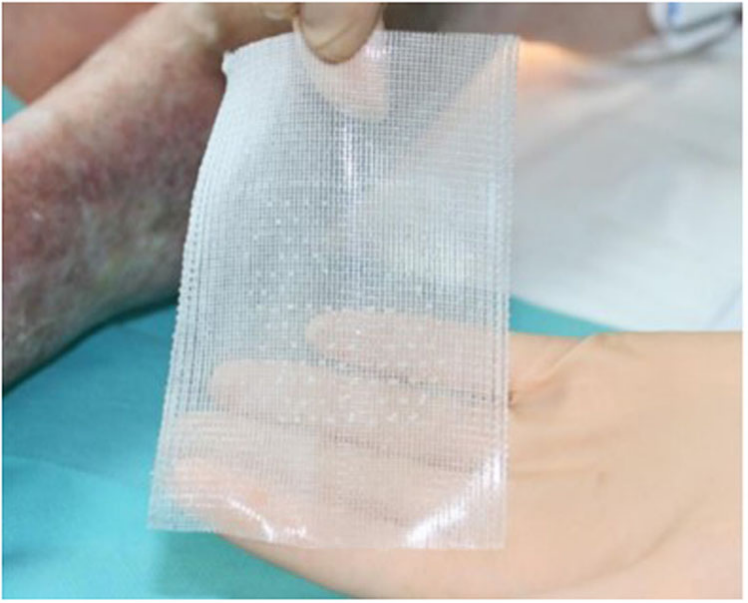
The harvested epidermal grafts transferred onto the wound using a non‐adhering silicone dressing.

During the time of wound bed preparation, the patient will be referred to the nurses specialized in wound healing to optimize the debridment of the wound. When the wound bed is deemed ready for grafting, patients will then be offered a patient information sheet for inclusion in the trial with a specific informed consent. An information sheet has been drawn up for patients. All wounds will be prepared per normal clinical practise to achieve a healthy granulating bed. Patients will undergo this procedure in a specific room without any local or general anesthesia. Before grafting, the wound will be cleaned using wound irrigation solution by the nurse and debrided if necessary. The device used was the CelluTome™ Epidermal Harvesting System (KCI, an Acelity company, San Antonio, TX) an automated epidermal harvesting system that produces an array of epidermal micro‐grafts). It consists of a control unit that creates and regulates the suction (negative pressure (−400 to −500 mmHg) and temperature (37°C to 41°C) required to raise the microdomes. It also includes a reusable suction head, which transmits negative pressure and heat from the control unit to the housing. The sampling box, a single‐use component of the system, is attached to the inside of the thigh by Velcro, after disinfection, +/− shave the donor site (generally the lateral or anterior surface of the thigh, homolateral to the grafted ulcer) (figure 1). This suction head of the CelluTome Epidermal Harvesting System will be applied to the thigh of the patient (donor site, generally the lateral or anterior surface of the thigh, homolateral to the grafted ulcer) for 40 min to harvest the epidermal graft. Only two types of heating plate are available (either 2.5 cm by 2.5 cm or 5 cm by 5 cm), which determines the maximum surface area of wounds that can be treated. The quality of dome formation could be assessed by visual inspection through the window of the box. Initially, the heating time was set at 40 min, but this could be increased depending on the effectiveness of the suction on the type of skin removed. If the epidermal domes were not raised, the time could be extended to 70 min. The harvested epidermal grafts will then be transferred onto the wound using a non‐adhering silicone dressing (Adaptic Touch®, Systagenix, Airebank Mills, Skipton, UK) (figure 2). The wound is then dressed with an absorbant dressing (Sorbact absorbant®, Inresa, Bartenheim, France), for wounds that are more exudative. The dressing will be fixed with a bandage, a cohesive strip without applying compressive force (Coheban® 3 M™, Cergy‐Pontoise, France). The donor site will be dressed with an adhesive hydrocellular dressing. The wound and donor site will be reviewed on day 8 (D8) ± 2 post‐grafting. An information sheet has been drawn up for the nursing teams to inform them of the procedure and the important principles of postprocedure monitoring, such as not opening the dressing until 8 days after the graft and not digging up the wound for 1 month after the procedure. In the case of very exudative wounds, the secondary absorbent dressing could be changed at D4, without touching the primary dressing.

Several grafts were possible. If several grafts were performed, a period of 2 months was required before repeating a procedure. The procedure was carried out by wound care nurses on an outpatient basis and transplant patients were monitored at D8, M1 and M3. Patients were informed about the use of their data and a specific research declaration was filed with the Clinical Research Department of Grenoble University Hospital on 30 June 2023.

## 5 Results

A total of 28 patients with chronic wounds were included in the study, 18 men and 10 women. All the inclusions were all consecutive, and no patient objected to the collection of their data.

**Figure 3:**
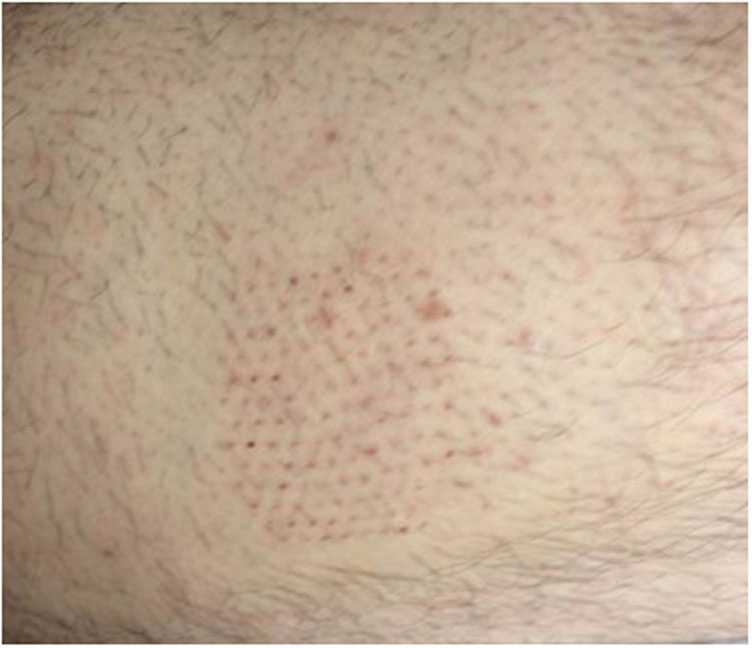
Graft scar on the donor site at the thigh at day 8.

The average age of the patients was 75.11 (16.24) mean (SD) years. All the wounds concerned by the procedure were located on the lower limb, with the exception of one post‐radiation dorsal wound. The main aetiologies of the chronic wounds were vascular: 23 “vascular” wounds were treated (82.2%), including 11 of venous origin (39.3%), 5 of arterial origin (17.8%) and 7 mixed ulcers (25%). The other aetiologies (17.8%) were post radiation (*n* = 1), post necrotizing fasciitis (*n* = 1) or related to stasis edema (*n* = 3). No side effects have been reported and there were no donor‐site scarring. The healing of the donor site was usually obtained in 8 days, as here on an illustration (Figure 3).

The duration of the ulcers was very heterogeneous, 22.7 (19.52) weeks, ranging from 4 months to 30 years, with an average duration of 6.4 (7.73) years. The vast majority of patients had a vascular history (arterial hypertension, type 2 diabetes, deep vein thrombosis, obesity) or factors contributing to delayed healing (Table 1). The average wound size on the day the first Cellutome® was applied was 18 (8.99) cm^2^.

**Table 1:** Caracteristic data of the patients with epidermal grafts.

Patient	Sex	Age	Localization of the wound	Aetiology of the wound	Aera in cm2	Duration of ulcer in years	Comorbidity	Number of cellutomes	Healed or reduction in area: 1	Effective analgesia 1/Ineffective analgesia 0	Follow‐up in months
									Unhealed: 0		
1	Male	36	lower limb	VLU	10	4	Thrombo‐embolic disease, post thrombotic syndroma	3	1	1	41
2	Female	97	lower limb	VLU	5	3	Venous insufficiency, rheumatoid arthrititis, systemic sclerosis	3	0		24
3	Male	94	lower limb	VLU	7	3	Thrombo‐embolic disease, systemic sclerosis	3	1		18
4	Female	91	lower limb	VLU	15	0.3	poliomyelitis, transtibial and toe amputation	2		0	90
5	Female	75	lower limb	VLU	1.8	6	Venous insufficiency, osteosynthesis of malleolus	2		1	8
6	Female	83	lower limb	Mixted ulcer	10.5	6	HTA	1		1	1
7	Male	75	lower limb	VLU	25	7	Obesity	2	0		27
8	Male	66	lower limb	Mixed ulcer	25	7	Systemic sclerosis	2	1		40
9	Male	74	lower limb	VLU	25	30	Post thrombotic syndroma, prot C deficiency	2	0		38
10	Male	90	lower limb	Post radiotherapy	7	3	Venous insufficiency	2	0	0	14
11	Female	82	lower limb	VLU	6.25	3	HTA, diabetis,	1	1		10
12	Male	95	lower limb	arterial limb ischemia	25	2	HTA, dyslipidemia, arterial limb ischemia	2		0	15
13	Male	83	dorsal	Post radiotherapy	12	1	Dorsal melanoma	6	1		30
14	Female	61	lower limb	Mixed ulcer	25	12	arterial limb ischemia and venous insufficiency	5		0	44
15	Male	93	lower limb	arterial limb ischemia	25	2	HTA, stroke, arterial limb ischemia	2	0	1	11
16	Female	82	lower limb	Mixed ulcer	25	3	HTA, rheumatoid arthrititis	2	0	1	52
17	Male	72	lower limb	arterial limb ischemia	25	1	Stroke, diabetic, HTA	1	1	1	23
18	Female	77	lower limb	arterial limb ischemia	11.2	7	arterial limb ischemia, HTA, rheumatoid arthrititis	1	0		24
19	Male	79	lower limb	VLU	0.75	29	Pulmonar embolism	1	1		16
20	Male	75	lower limb	Mixed ulcer	8.75	3	arterial limb ischemia, coronaropathy	1	0		16
21	Male	77	lower limb	VLU	6.1	2	Post thrombotic syndroma	2	1		12
22	Female	50	lower limb	arterial limb ischemia	15	1	sickle cell disease	2	1		13
23	Male	79	lower limb	Mixed ulcer	35	6	Coronaropathy, pulmonar embolism, asthma, HTA, diabetic, stroke, rheumatoid arthrititis	1	1		2
24	Male	56	lower limb	Post fasciite	10	15	tabacco	1	0		4
25	Male	90	lower limb	VLU	5.4	1	Dibetic, post thrombotic	2	0		4
26	Female	42	lower limb	Post radiotherapy	3.5	0.3	hodgkin's disease, systemic sclerosis, osteosarcoma	1	0		2
27	Male	54	lower limb	Mixted ulcer	8	3	HTA, kidney transplant	1	0	0	34
mean (standard deviation)		75.11 (16.25)			18(8.99)	6.4 (7.73)		2 (1.21)			22.70 (19.52)

The number of Cellutome® was 2 (1.21) per patient (range 1‐6). Patient follow‐up averaged 22.70 (19.52) weeks (range 1–90).

The indications of the grafts could be either for analgesic purposes only (*n* = 2), or for healing (*n* = 16) or with these two objectives combined (*n* = 10). For the 16 patients whose objective was healing, only 15 could be analyzed with a patient lost by 1 month. Of these 15 patients, 4 were healed after 1 month after the last Cellutome®, or 26.6%. For the analgesic purpose, only one patient no longer had pain in post transplant.

**Figure 4:**
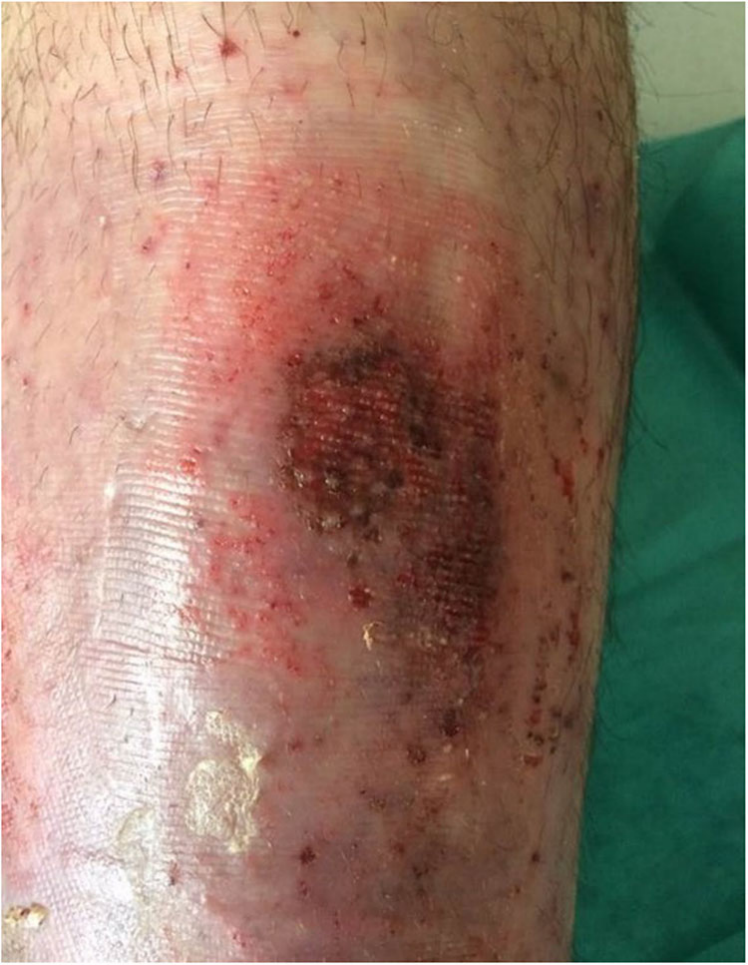
Epidermal islets at day 8 on a post‐thrombotic ulcer.

**Figure 5:**
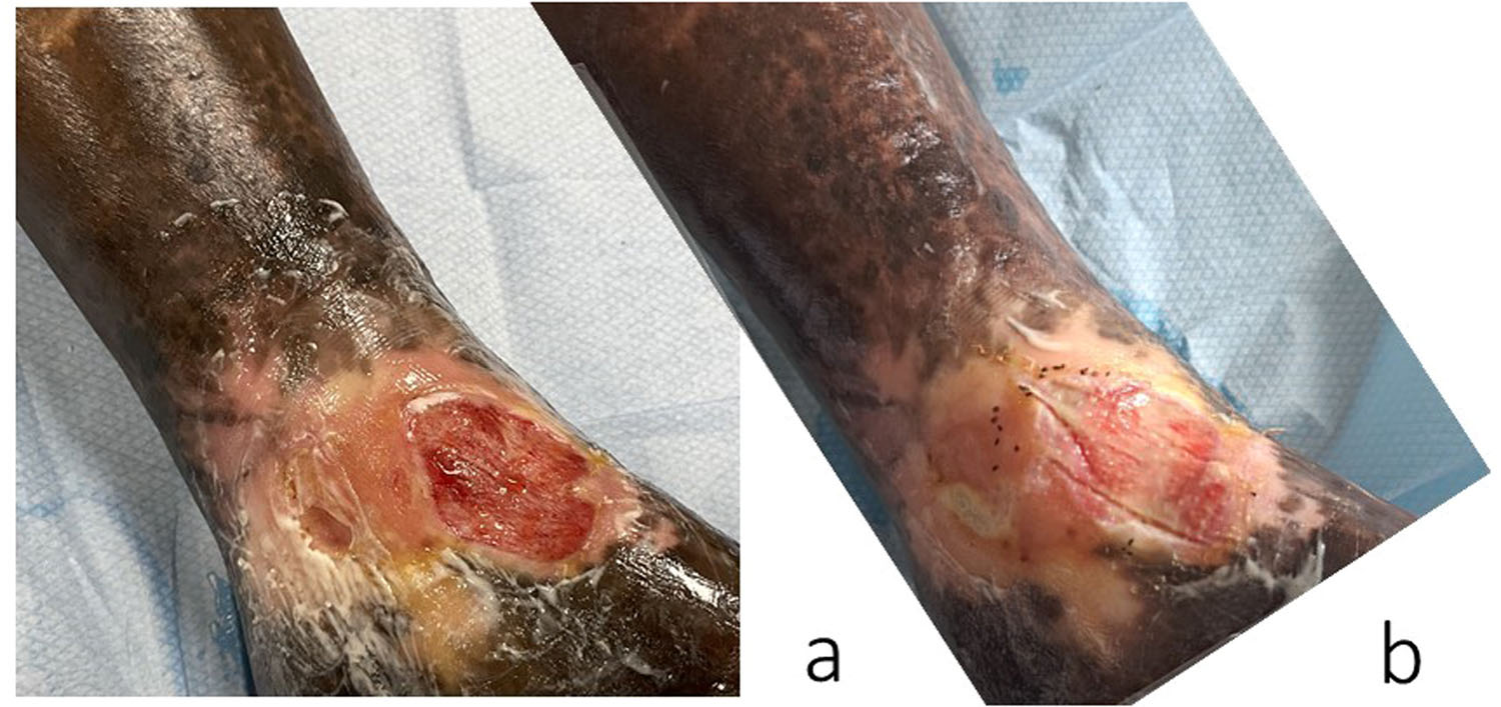
(a) ulcer of a sickle cell patient (b) day 8 ulcer healing.

The result in terms of healing is difficult to assess since some patients have healed between 2 Cellutome ® procedures but had recurred between 2 transplants (Figure 4). Some wounds considered difficult to heal as here an ulcer of a sickle cell patient who healed after only 8 days after the transplant (Figure 5).

## 6 Discussion

This retrospective study demonstrates the potential value of epidermal grafting as a treatment for seams in hard‐to‐heal wounds [3]. Certain more specific indications seem to be emerging, such as vitiligo [4], second degree burns [5] or epidermolysis bullosa [6]. Nevertheless, the study is marked by many of the biases usually found in the evaluation of treatments for chronic wounds, especially in retrospective studies (patients lost to follow‐up, poorly reproducible evaluation criteria and missing data). The main pitfall is that wound surface areas have not been calculated. Thus, for patients who have not healed, the evolution of wounds in terms of surface area cannot be given accurately. The sample size, despite the numerous biases, particularly the retrospective nature, is highly representative of the center's clinical practice. The traceability of the medical device ensures that all patients are included.

Nevertheless, epidermal grafts offer a number of advantages in the field of wound healing, particularly in difficult, recalcitrant and multifactorial wounds, as in our series [4, 7, 8]. The ease with which these grafts can be performed (broad aetiological indications [9], ambulatory nature, no need for a complex technical platform, no need for anesthesia) makes them accessible to a large number of patients. The procedures can be carried out by specialized nurses, without the need to involve medical staff. Performing this type of procedure is painless for the patient, which makes it easier for them to adhere to the procedure and makes it possible to perform several procedures iteratively. The speed with which the grafts can be put in place, without the need for an operating theater [10], means that the procedure can be performed quickly, particularly in cases of painful wounds (e.g. necrotic angiodermatitis). The technique is particularly suitable for outpatients [1]. What's more, the system is inexpensive [11, 12, 13]. There is very little discomfort for the patient and the graft scar is extremely discreet. Epidermal micrografts formed at the dermal‐epidermic junction, brings basal layer keratinocytes, melanocytes, basement membrane‐specific collagen type IV, viable basal cells actively secreted key growth factors important for modulating wound healing responses [10].

Despite these advantages, the results of epidermal grafts in terms of efficacy are mixed and partial. We did not find any comparative studies between the different superficial grafts (epidermal, pellet or mesh). Only one randomized study was found comparing Split‐thickness skin grafting and Epidermal grafting favoring Split‐thickness skin grafting [2]. This type of study would be difficult to set up because the indications are not identical with the limited surface and deep indications for epidermal grafts.

This procedure undoubtedly deserves to be better known and more widely used to optimize the experiments, particularly with regard to heating times. The recommended heating time is at least 40 min, but in practice it seems important to adapt to the clinic and to extend the procedure if the epidermal domes do not appear. The hypotheses discussed would be that the heating time required could vary according to the skin thickness (sampling site chosen but above all the age of the patient and/or the patient's skin hydration level). These aspects must be taken into consideration to ensure the success and efficacy of epidermal grafts.

In conclusion, despite the advantages of epidermal grafts in terms of ease of implementation and speed, as well as their minimally invasive and painless nature, it is essential to remain aware of the limitations of this procedure and to take potential drawbacks into account to ensure optimal results for patients.

## Author Contributions

**S. Bruyeres:** conceptualization, data curation, formal analysis, investigation, methodology, project administration, resources, supervision, validation, visualization, writing – original draft, writing – review and editing. **G. Dumas:** data curation, investigation, methodology, validation, visualization, writing – review and editing. **N. Zenati:** resources, validation, writing – review and editing. **S. Blaise:** conceptualization, data curation, formal analysis, investigation, methodology, project administration, resources, supervision, validation, visualization, writing – original draft, writing – review and editing.

## Conflicts of Interest

All authors have read and approved the final version of the manuscript. Pr S. Blaise had full access to all of the data in this study and takes complete responsibility for the integrity of the data and the accuracy of the data analysis. Pr S. Blaise affirms that this manuscript is an honest, accurate, and transparent account of the study being reported; that no important aspects of the study have been omitted; and that any discrepancies from the study as planned have been explained.

## Transparency Statement

The lead author S. Blaise affirms that this manuscript is an honest, accurate, and transparent account of the study being reported; that no important aspects of the study have been omitted; and that any discrepancies from the study as planned (and, if relevant, registered) have been explained.

## Data Availability

The authors confirm that the data supporting the findings of this study are available on specific request.
